# Resilience of compound action potential peaks to high‐frequency firing in the mouse optic nerve

**DOI:** 10.14814/phy2.15606

**Published:** 2023-02-17

**Authors:** Amy J. Hopper, Hana Beswick‐Jones, Angus M. Brown

**Affiliations:** ^1^ School of Life Sciences University of Nottingham Nottingham UK; ^2^ Department of Neurology, School of Medicine University of Washington Seattle Washington USA

**Keywords:** action potential, potassium, sodium

## Abstract

Action potential conduction in axons triggers trans‐membrane ion movements, where Na^+^ enters and K^+^ leaves axons, leading to disruptions in resting trans‐membrane ion gradients that must be restored for optimal axon conduction, an energy dependent process. The higher the stimulus frequency, the greater the ion movements and the resulting energy demand. In the mouse optic nerve (MON), the stimulus evoked compound action potential (CAP) displays a triple peaked profile, consistent with subpopulations of axons classified by size producing the distinct peaks. The three CAP peaks show differential sensitivity to high‐frequency firing, with the large axons, which contribute to the 1st peak, more resilient than the small axons, which produce the 3rd peak. Modeling studies predict frequency dependent intra‐axonal Na^+^ accumulation at the nodes of Ranvier, sufficient to attenuate the triple peaked CAP. Short bursts of high‐frequency stimulus evoke transient elevations in interstitial K^+^ ([K^+^]_o_), which peak at about 50 Hz. However, powerful astrocytic buffering limits the [K^+^]_o_ increase to levels insufficient to cause CAP attenuation. A post‐stimulus [K^+^]_o_ undershoot below baseline coincides with a transient increase in the amplitudes of all three CAP peaks. The volume specific scaling relating energy expenditure to increasing axon size dictates that large axons are more resilient to high‐frequency firing than small axons.

## Graphical abstract

High‐frequency firing in the mouse optic nerve results in greater Na^+^ accumulation in smaller axons than larger axons, whereas the interstitial [K^+^]_o_ increase in limited. Post‐stimulus [K^+^]_o_ transiently falls below baseline resulting in augmented CAP amplitude.

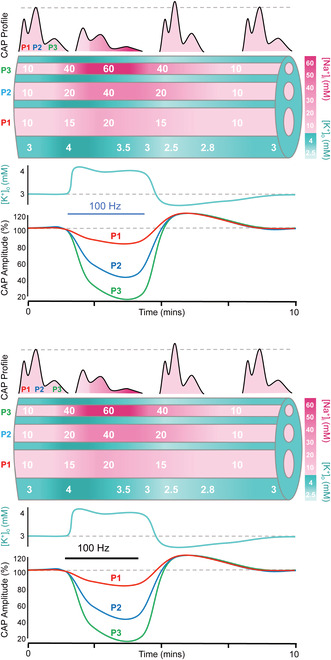

## 1 INTRODUCTION

In the mammalian central nervous system (CNS), the diverse population of axons present reflects a wide range of axon sizes (0.1 to 10 μm, Zang & Marder, 2021) coupled with the selective presence of myelin. Although increasing size and myelin bestow axons with the benefits of faster conduction (Nicholls et al., 2012), the majority of mammalian corpus callosum axons are unmyelinated (Wang et al., 2008), and the median axon diameter range is only 0.6 to 1 μm in human cortical white matter (Liewald et al., 2014). In the mouse optic nerve (MON), a central white matter tract that encodes visual information, all axons are myelinated in the adult animal (Edgar et al., 2010). The diameter range is 0.2 to 2.7 μm, the median value is 0.7 μm and the distribution is skewed with small axons dominant (Allen et al., 2006; Perge et al., 2012).

Since axon energy expenditure increases with size, the small diameter of most CNS axons constrains their conduction velocity (Karbowski, 2007) and firing frequency (Perge et al., 2009), from which the information transfer rate is computed (Koch et al., 2006). The frequency encoding typical of sensory pathways, which contain a wide range of axons sizes, relies of action potential fidelity over long distances. Although large axons conduct at high frequency, they are less efficient per unit volume than smaller axons, which use less energy to conduct more bits/s (Perge et al., 2009). Evolutionary pressure favors small axons; thus, large axons must justify their excessive energy consumption. The relatively few large axons in MON are proposed to conduct at high frequency (30 Hz), where the basal firing rate of small axons is 5 Hz (Perge et al., 2009). To achieve this, the large axons must be fatigue resistant, an important consideration given the significant trans‐membrane ion movements that accompany action potential firing at high frequency. Action potential conduction involves Na^+^ influx leading to nodal Na^+^ accumulation (Gerkau et al., 2019), followed by K^+^ efflux, which collects in the interstitial fluid (Ransom et al., 2000; Rasmussen et al., 2020). The energy required to restore ion gradients is dependent upon firing frequency (Harris & Attwell, 2012). A quantitative model predicts action potential conduction consumes 47% of available energy in gray matter (Attwell & Laughlin, 2001). A considerable volume (30%) of the MON is occupied by astrocytes (Perge et al., 2009), which efficiently buffer interstitial K^+^ via the energy dependent Na^+^K^+^ATPase (NKA) and Kir4.1 channels, such that [K^+^]_o_ is maintained below a ceiling level (Ransom et al., 2000), allowing fidelity of sustained action potential conduction. Computational studies based on surface area to volume relationships of nodal Na^+^ channel density, NKA expression and axoplasmic mitochondrial density proposed intra‐axonal accumulation of Na^+^ as a likely cause of action potential decrease at high firing frequencies, with smaller axons more susceptible than large axons (Perge et al., 2009; Zang & Marder, 2021).

The purpose of this paper was to use the MON as model with which to study the energy constraints on information transmission, and the trade offs that neural systems make between energy consumption, speed, and information density. The three distinct peaks of the stimulus evoked CAP are formed from contributions of three populations of axons classified by size. Manipulation of the Nernst equation (Hopper et al., 2022), where CAP peak amplitudes were assumed to follow changes in E_Na_ resulting from decreases in aCSF [Na^+^], allowed predictions of the intra‐axonal [Na^+^]_i_, satisfactorily accounting for the frequency dependent differential decrease in CAP peak amplitudes. The interstitial [K^+^]_o_ does not rise to levels capable of sufficiently depolarizing the axonal membrane potential to levels which would lead to Na^+^ channel inactivation during high‐frequency firing, but its frequency dependent increase indicates a role in alerting astrocytes to on‐going neuronal activity (Sotelo‐Hitschfeld et al., 2015).

## 2 METHODS

### 2.1 Ethics statement

All experiments were approved by the University of Nottingham Animal Care and Ethics Committee, were carried out in accordance with the Animals (Scientific Procedures) Act 1986 under appropriate authority of establishment, project and personal licenses, and conform to the principles and regulations described in the Editorial by Grundy (Grundy, 2015). Experiments were performed on male CD‐1 mice (weight 28–35 g, corresponding to 30–45 days of age) purchased from Charles River Laboratories. Mice were group housed with ad libitum access to food and water and maintained at 22–23°C on a 12 h:12 h light–dark cycle. Mice were killed by Schedule 1 cervical dislocation; death was confirmed by permanent cessation of the circulation. A total of 78 mice were used, with both optic nerves used for recordings in separate chambers. In total, 119 optic nerve recordings were made.

### 2.2 Dissection and recording environment

Male CD‐1 mice were killed by cervical dislocation and then decapitated. The optic nerves were exposed by lifting the cerebral hemispheres, and the nerves (5–7 mm long) were cut at the optic chiasm and behind the orbit. The optic nerves were gently freed from their dural sheaths and placed in an interface perfusion chamber (Medical Systems; Stys et al., 1991). MONs were maintained at 37°C using a TC‐202A bipolar temperature controller (Digitimer) and perfused with artificial cerebrospinal fluid (aCSF) that contained (in mM): 153 Na^+^, 3 K^+^, 2 Mg^2+^, 2 Ca^2+^, 143 Cl^−^, 26 HCO_3_^−^, 1.25 HPO_4_^2−^, and 10 glucose. In experiments where Na^+^ was reduced equimolar choline chloride was added, and when K^+^ was altered an equimolar substitution in Na^+^ was made to maintain osmolarity. The aCSF was bubbled with a gas mixture (95% O_2_: 5% CO_2_) to maintain pH at 7.45. Tissue was oxygenated by a humidified gas mixture (95% O_2_: 5% CO_2_) that flowed over its surface. A stimulating suction electrode back‐filled with aCSF was attached to the proximal end of the nerve. A recording electrode filled with aCSF was attached to the distal end of the nerve to record the CAP, which was evoked by a 125% supramaximal stimulus (30 μs in duration: a Grass S88 stimulator connected to two SIU5 isolation units in parallel capable of delivering two independently controllable pulses). CAPs were recorded from a suction electrode attached to the distal end of the nerve connected to an SR560 low noise preamplifier (Stanford Research Systems): The signal was amplified up to 1000× and filtered at 30 kHz. The differential output was fed into a HumBug Noise Eliminator (Digitimer Ltd) to remove mains frequency noise (50 Hz). The signal was acquired via an Axon Digidata 1550B using Clampex 11.3 (Molecular Devices Ltd). Nerves were allowed to equilibrate for 30 min before recording commenced. The MON was stimulated at a baseline frequency of 1 Hz unless indicated. Increases in stimulus frequency were applied in continuous, uninterrupted episodes of 10 s, where the CAP recorded was the average of all CAPs within this period (e.g., for a 2‐min period of stimulus 12 sequential episodes were imposed).

### 2.3 K^+^ sensitive microelectrodes

K^+^‐sensitive microelectrodes were made with double‐barreled piggyback glass (WPI, PB150F‐6) as previously described (Borrelli et al., 1985), with slight modifications. Electrodes were broken back to a tip diameter of >5 μm by gentle agitation against a scalpel blade under a light microscope (×10). The tip of the ion‐sensitive barrel was back‐filled with N,N‐dimethyltrimethylsilyamine (41716; SigmaAldrich, Merck Life Science UK Ltd) and baked at 160°C for 1 h (Ambiano mini oven, Aldi). The indifferent barrel was back‐filled (Microfil 34 gauge tip, WPI) with 150 mM NaCl, 20 mM HEPES adjusted to pH 7.4 with 1 M NaOH. The ion‐sensitive barrel was back‐filled (Microfil 28 gauge tip, WPI) with 100 mM KCl, 20 mM HEPES adjusted to pH 7.2 with 1 M NaOH. The ion‐sensitive barrel was filled at the tip by front suction with a short (100–400 μm) column of K^+^ sensitive liquid ion sensor (K ionophore I Cocktail B, 99373, SigmaAldrich). The ion‐sensitive microelectrode was connected to an HS‐2 ×0.0001 MU headstage. The indifferent (reference) electrode was connected via a HS‐2A ×0.1 LU headstage. Both headstages were connected to an Axoprobe 1B amplifier (Molecular Devices Ltd). The reference signal was subtracted from the ion‐sensitive signal and amplified 10× then fed into a HumBug Noise Eliminator (Digitimer Ltd) to remove mains frequency noise (50 Hz). The signal was acquired via a MiniDigi 1A using Axoscope 11 (Molecular Devices) at 10 Hz. Electrodes were calibrated in a solution containing 120 mM NaCl and 20 mM HEPES, with KCl at concentrations of 3 mM, 9 mM or 30 mM. A motorized manipulator (EC1 60‐0577 control unit with EC1 60‐0571 standard motorized control manipulator: Harvard Apparatus) was used for placement of the K^+^ sensitive microelectrode in the nerve. All electrodes were individually calibrated and only those showing stable, near Nernstian responses (i.e., 50–60 mV) to decade changes in [K^+^] were used for experimental measurements. Electrodes were recalibrated after each experiment and data from electrodes with greater than a 5 mV deviation in response to decade changes in [K^+^] were discarded. The average between the initial and final calibrations was used to evaluate experimental data. The [K^+^]_o_ was estimated using the following rearrangement of the Nernst equation:

(1) $${\left[K\right]}_{o}=3\;\mathrm{mM}\;x\;10^{\left(\frac{V}{\text{slope}}\right)}$$

where *V* is the differential output from the amplifier in mV and slope is the electrode response to a decade change in K^+^ from 3 to 30 mM in mV. Post experimental calibrations and data manipulations were carried out using Microsoft Excel (Microsoft).

### 2.4 Transmission electron microscopy (TEM)

Mouse optic nerves were dissected as described above, laid out on cardboard and fixed in 2% glutaraldehyde and 2% paraformaldehyde solution in 0.2 M phosphate buffer overnight and post‐fixed in 1% osmium tetroxide for 30 min. They were dehydrated in a graded ethanol series and embedded in Transmit low viscosity resin (TAAB). Semi thin sections were cut at 0.5 µm, stained with toluidine blue and photographed using a Leica DM400B light microscope with color digital camera and Openlab darkroom software. Ultrathin sections (70–90 nm) were prepared using a Reichert‐Jung Ultracut E ultramicrotome and mounted on 100 hexagonal copper grids. They were contrasted using uranyl acetate and lead citrate and viewed using a JEOL 1010 TEM operated at 80 kV with digital image acquisition.

### 2.5 Data analysis

**FIGURE 1:**
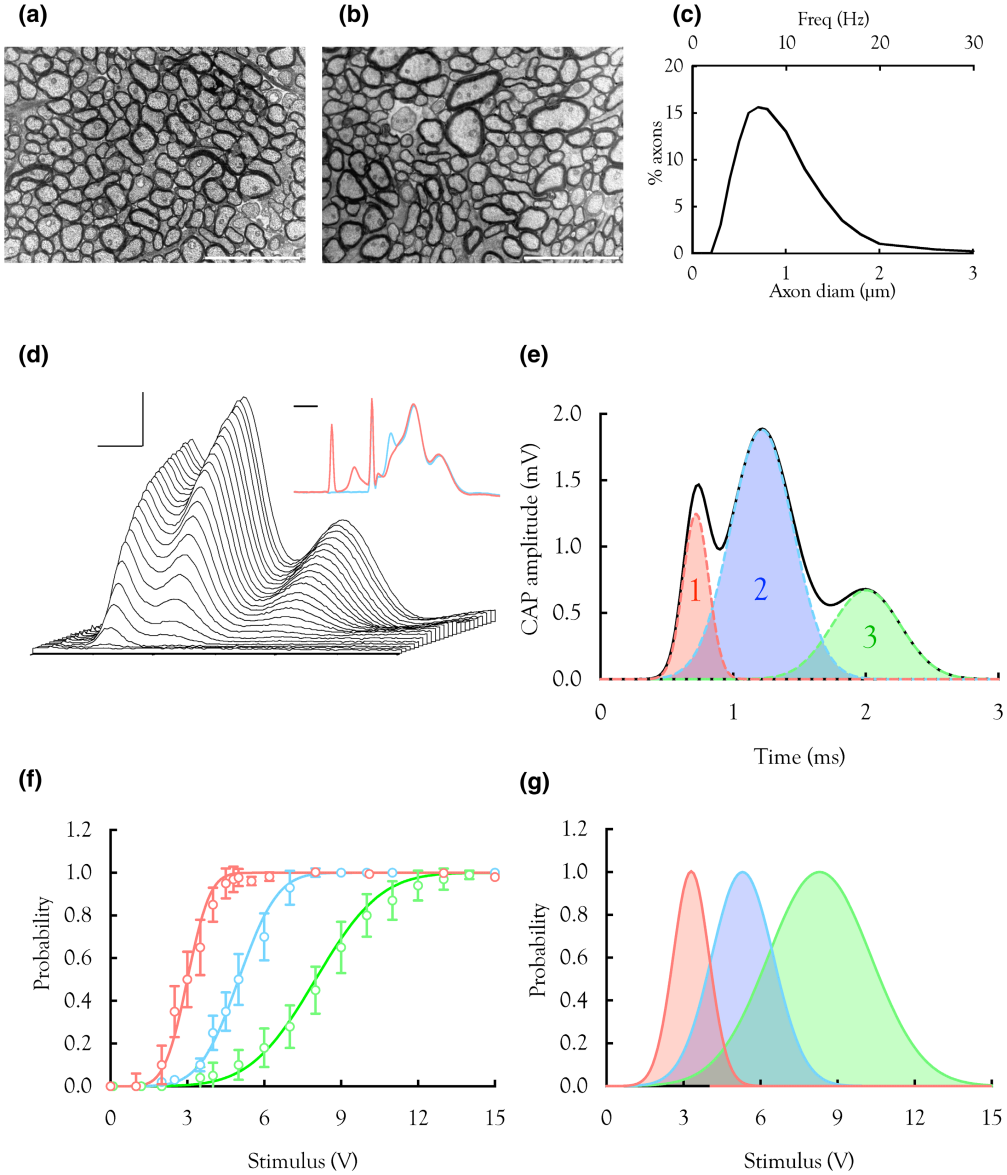
The CAP recorded from adult MON. (a,b) Low power electron micrographs of transverse sections of adult MON. The majority of myelinated axons are less than 1 μm in diameter, although there are a few conspicuously larger axons present ((b) – upper centre). Scale bars 5 μm. (c) Frequency histogram of axons in rodent optic nerve, whose diameters range from 0.2 to 3 μm, with the median value of 0.7 μm (lower x‐axis), superimposed with computed firing frequency related to axon size (upper x‐axis) (Perge et al., 2009). (d) A waterfall image of CAP recruitment with increasing stimulus voltage depicted on the Z‐axis, the stimulus amplitude increasing towards the rear of the stack, showing sequential recruitment of the 1st peak, followed by the 2nd then 3rd peak. The CAP peaks asymptote as the stimulus is increased. Scale bars are 1 ms and 1 mV. Inset. A double pulse protocol was employed in which the first stimulus was of sufficient strength to recruit only the 1st peak. A subsequent pulse of supramaximal strength 1 ms later recruited only the 2nd and 3rd peaks (red trace). The blue trace shows the control CAP in the absence of a 1st stimulus pulse (*n* = 6). Scale bar = 0.5 ms. (e) CAP (black bold line) evoked by a supramaximal stimulus, can be accurately described as the sum of 3 overlapping Gaussian distributions. Peak areas are 0.87 ± 0.06 mV.ms for the 1st peak, 2.80 ± 0.41 mV.ms for the 2nd peak, and 2.17 ± 0.26 mV.ms for the 3rd peak (*n* = 16). In such fits, the *R* ^2^ value typically exceeds 0.98. The stimulus artifact has been removed to improve clarity. The 2nd peak overlaps the 1st peak, but the 2nd and 3rd peak amplitudes are unaffected by neighboring peaks. The 1st peaks are represented by red, the 2nd by blue and the 3rd by green, a color scheme used in the subsequent figures. (f) Fitting traces like those illustrated in (d) with Gaussian fits of individual peaks, produces data that can be plotted as individual peak areas versus stimulus voltage as a cumulative probability function, which yields the following values for mean ± SD; 1st peak 3.0 ± 0.7 V, 2nd peak 5.1 ± 1.2 V, 3rd peak 7.9 ± 2.0 V (*n* = 4 for each peak). The 1st peak displayed the lowest threshold for recruitment and the steepest slope. (g) The parameters resulting from the curve fits in (f) can be replotted as Gaussian probability functions, whose profiles closely resemble the fits of the individual peaks illustrated in (e).

The triple peaked CAP was analyzed using Clampfit 11 (Molecular Devices). Measurement of the amplitudes of the individual peaks was complicated as the 2nd CAP peak overlapped the 1st CAP; thus, the amplitude of the 1st CAP peak contains considerable 2nd peak contribution. The 2nd peak and 3rd CAP peaks do not significantly superimpose; thus, measurement of their amplitudes is straightforward (Figure 1e). The 1st peak area was measured using a Gaussian fit and converted to an amplitude to eliminate interference from the 2nd peak, according to

(2) $$\mathrm{Amp}=\frac{\text{Area}}{w\;x\;\sqrt{\frac{\pi }{2}}}$$

where *w* is the width of the peak.

The NORMDIST function in Microsoft Excel (Microsoft Corp) was used to fit Gaussian distributions in Figure 1g and their integrals (Figure 1f).

**FIGURE 2:**
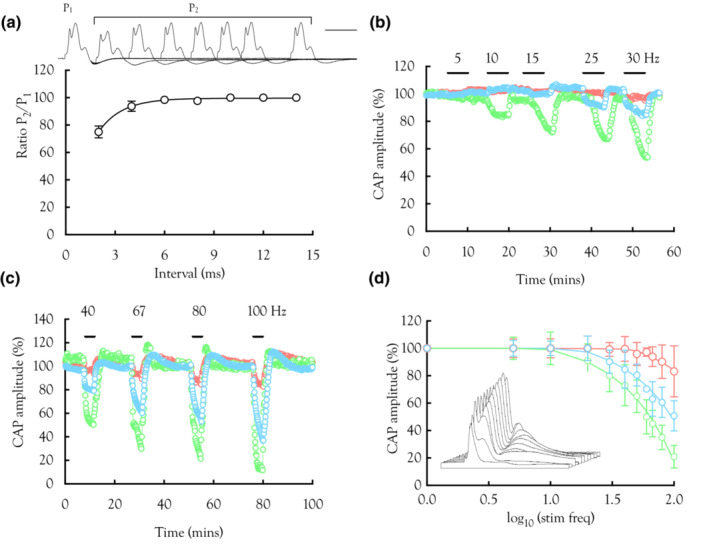
Increased firing frequency reduces CAP peak amplitudes. (a) Inset trace shows superimposed CAPs evoked by a double pulse protocol with the 2nd pulse (P_2_) occurring at decreased inter‐pulse intervals from the 1st (P_1_). Main graph: The ratio of P_2_/P_1_ plotted for the 2nd peak against the inter‐pulse interval shows a decreased P_2_ peak when the inter‐pulse interval is below about 5 ms. Scale bar 2 ms. Data fit by Equation 3 where *k* = 0.71 ms^−1^. All peaks were affected to the same extent (One‐way ANOVA *F*(2,15) 0.02, *p* = 0.92, *n* = 7). (b) Low‐frequency stimulus for 4 min (>40 Hz) from a representative individual experiment illustrates the sensitivity of the 3rd peak and the resilience of the 1st peak. (c) The effect was exacerbated with high‐frequency firing in a separate MON. (d) Plot of CAP peak amplitudes versus stimulus frequency (*n* varies from 4 to 9). Data were fit with a discontinuous curve (Equation 4) with the break point calculated as described in the section 2: Methods. The values were 45.4 Hz for the 1st peak, 15.6 Hz for the 2nd peak and 8.9 Hz for the 3rd peak. Inset: sequential plot of CAPs plotted every 10 s from a MON during 100 Hz stimulus. CAPs plotted in sequential order with the CAPs recorded earliest towards the rear of the stack (stimulus artifacts have been removed for clarity).

An exponential function of the form *y* = 1 – *e* ^−kt^ (Equation 3) was used to fit the data in Figure 2a.

We used a novel curve fitting routine, where the data illustrated in Figure 2d were fit by two straight lines. The first line is horizontal (i.e., slope = 1) denoting the range of frequencies at which action potential amplitude is fully maintained. A negative slope in the second line indicates CAP amplitude decline (de Levie, 2004; Gallant, 1977). The data were described by the function *y* = *a*_0_ + a_1_c + *b*_0_ + b_1_c (Equation 4) with Microsoft SOLVER used to estimate the branch point, which occurs at the intercept of the two lines, denoting the frequency above which axons cannot follow. The data illustrated in Figure 2d were logarithmically transformed to allow description by linear fits.

**FIGURE 3:**
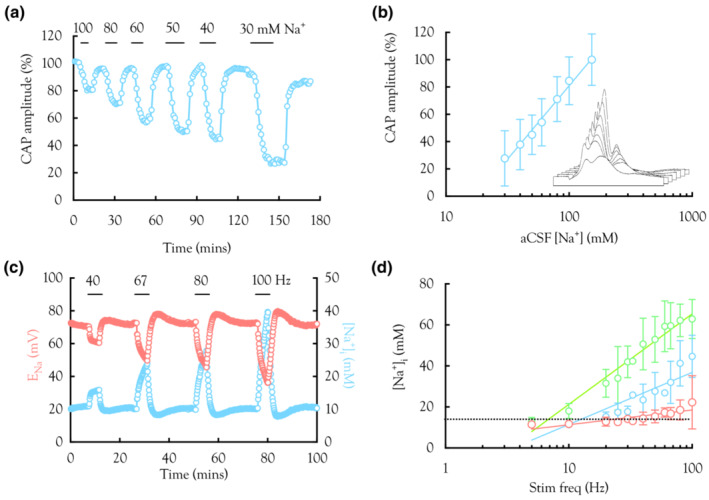
Reducing aCSF [Na^+^] attenuates CAP amplitude. (a) Representative individual experiment where reduction in aCSF [Na^+^] caused a reduction in the amplitude of all CAP peaks. The effect was identical across all peaks, and the 2nd peak was used for illustrative purposes (One‐way ANOVA *F*(2,20) 0.016, *p* = 0.98). The horizontal bars indicate the duration of exposure to low [Na^+^] aCSF. (b) Summary plot of 2nd peak CAP amplitude versus aCSF [Na^+^], which varied from 30 mM to control level of 153 mM (*n* varies from 14 to 17). The log linear fit is described by *y* = 46.4 ln (*x*)–133.1. Inset illustrates sequential effects of lowering aCSF [Na^+^] from 153.25 to 100, 80, 70, 60, 50, 40, and 30 mM where CAPs plotted in sequential order from highest to lowest [Na^+^]. (c) This graph is based on the 2nd peak data (blue) illustrated in Figure 2c. The CAP amplitude was converted to E_Na_ (red ‐ left y‐axis) based on the relationship described in the Methods: section 2. This was then used to calculate the corresponding [Na^+^]_i_ (blue ‐ right y‐axis). (d) Summary of data when predicted [Na^+^]_i_ was plotted versus stimulus frequency (*n* varies from 14 to 17). Based on Equation 10, the intercept for the fits when [Na^+^]_i_ was 15 mM (horizontal dotted line) was 32.3 Hz for the 1st peak, 13.8 Hz for the 2nd peak, and 7.2 Hz for the 3rd peak.

The relationship illustrated in Figure 3b was CAP = 106.9 x log_10_[Na^+^]_o_–133.1, rearranged as

(5) $${\left[{\mathrm{Na}}^{+}\right]}_{o}=10^{\left(\frac{\mathrm{CAP}+133.1}{106.9}\right)}$$

when solving for [Na^+^]_o_.

The Nernst equation can be expressed as

(6) $${\left[{\mathrm{Na}}^{+}\right]}_{o}=10\;x\;10^{\left(\frac{E_{\mathrm{Na}}}{61.5}\right)}$$

when [Na^+^]_i_ is 10 mM (Ransom et al., 2000), which allows Equations 5 and 6 to be combined as simultaneous equations, providing the following expression for *E*_Na_:

(7) $$E_{\mathrm{Na}}=0.57\;x\;\mathrm{CAP}+15.1$$

from which [Na^+^]_i_ can be estimated from the Nernst equation

(8) $${\left[{\mathrm{Na}}^{+}\right]}_{i}=\frac{153}{10^{\left(\frac{E_{\mathrm{Na}}}{61.5}\right)}}$$

assuming [Na^+^]_o_ was 153 mM and was unchanged during stimulus.

The aCSF [K^+^] dose response curve was described by the function

(9) $$\mathrm{CAP}=1+e^{\left(\frac{K_{50}-\left[K\right]}{\text{slope}}\right)}$$

**FIGURE 4:**
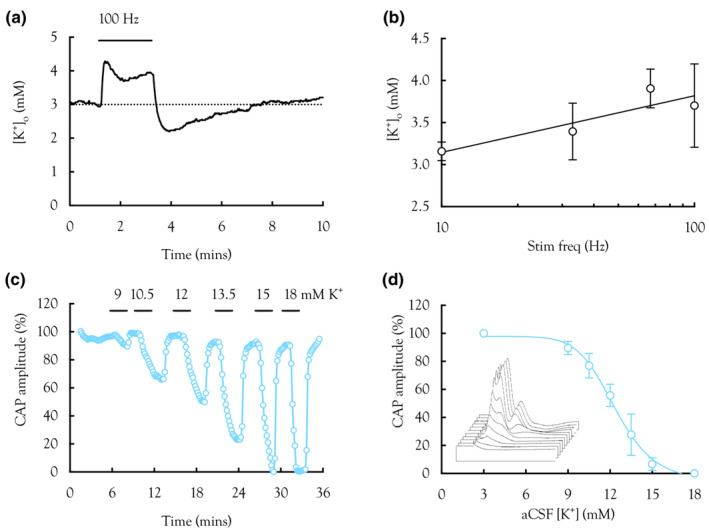
High‐frequency stimulus and [K^+^]_o_. (a) A representative trace illustrates the effect of a 2 min 100 Hz pulse on [K^+^]_o_ recorded using K^+^ sensitive microelectrodes. [K^+^]_o_ rose rapidly to a peak, stabilized to a plateau, before undershooting the baseline on cessation of the stimulus, followed by a slow relaxation towards baseline. (b) Summarized plot of peak [K^+^]_o_ versus stimulus frequency for 2 min stimulus (*n* = 5), described by the log linear relationship *y* = 1.84 ln (*x*)–1.1. (c) Representative recording from a MON exposed to aCSF in which [K^+^] was separately increased from a baseline of 3 to 18 mM. The horizontal bars indicate the duration of exposure to K^+^; absence of bars indicates bathing in 3 mM [K^+^]. (d) Summarized plot of CAP amplitude versus [K^+^]_o_, illustrating the CAP starts to fall at about 9 mM and is completely lost by 15 mM, [K^+^], with a half maximum effect at 12.3 mM and slope of 9 (*n* = 5: Equation 9). Inset illustrates sequential effect of increasing [K^+^] from 3 mM to 9, 10.5, 12, 13.5, 15, and 18 mM, where CAPs are plotted in sequential order with the highest concentrations towards the rear of the stack.

**FIGURE 5:**
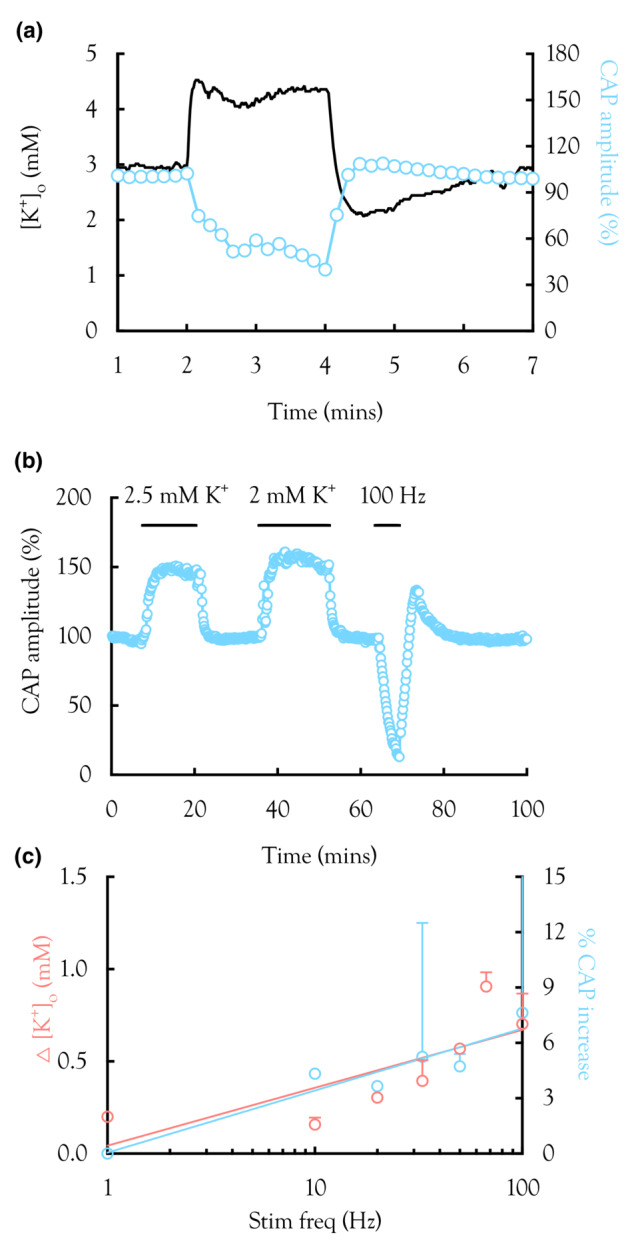
[K^+^]_o_ undershoot and CAP amplitude. (a) Simultaneous recordings of the CAP and [K^+^]_o_ from the same nerve. In response to 2 min of 100 Hz stimulus, the [K^+^]_o_ was as described in Figure 4a. However, the CAP fell more slowly. On cessation of stimulus the CAP increased briefly, overshooting its baseline amplitude before relaxing towards baseline. The profile of the CAP recovery was mirrored by the undershoot of the post‐stimulus [K^+^]_o_ response. (b) Representative experiment (*n* = 4) in which aCSF [K^+^] was decreased to 2.5 mM then 2 mM, causing an increase in the 2nd peak amplitude. A 5‐min 100 Hz stimulus caused the peak to fall then overshot baseline briefly on recovery. (c) Summary of data from experiments like those illustrated in A in which [K^+^]_o_ and the CAP were recorded simultaneously. The magnitude of the [K^+^]_o_ undershoot (blue ‐ left y‐axis) was plotted versus stimulus frequency with CAP overshoot (red ‐ right y‐axis), overlaid and scaled for optimal overlap (*n* = 4).

Log linear relationships of the form *y* = *a* ln (*x*) + *b* (Equation 10) were used to describe data in Figures 3b,d, 4b and 5c.

Data were organized in Microsoft Excel. Graphs were created in GraphPad Prism, and data are presented as mean ± standard deviations (SD). One‐way ANOVA was used to determine inter‐peak differences when varying aCSF [Na^+^] or [K^+^].

## 3 RESULTS

### 3.1 The stimulus evoked compound action potential recorded from adult MON

The adult MON comprises about 25,000 myelinated axons (Allen et al., 2006; Edgar et al., 2010). The transverse section illustrated in Figure 1a shows numerous closely abutting small axons separated by a narrow interstitial space. However, a few large axons are present (Figure 1b). An idealized frequency histogram of axon size coupled with computations of firing frequency representative of rodent optic nerve axons shows a skewed relationship with axon diameter range from 0.2 μm to about 3 μm, corresponding to firing frequencies of 5–30 Hz, respectively (Figure 1c; Perge et al., 2009). The stimulus evoked CAP comprises three distinct but overlapping peaks, with increasing stimulus sequentially recruiting the 1st peak, the 2nd peak, then the 3rd peak (Figure 1d). We imposed a double pulse protocol to confirm that the axons contribute only to one peak, that is, there is no rebound firing where an axon can fire twice in rapid succession (Erlanger & Gasser, 1937). An initial conditioning pulse, of sufficient strength to recruit only the 1st peak, was followed by a supramaximal test pulse 1 ms later, which recruited only the 2nd and 3rd peaks. In the absence of the conditioning pulse, the test pulse elicited the control triple peaked CAP (Figure 1d: inset). The profile of the CAP is accurately fit as the sum of three Gaussian distributions (Figure 1e). By fitting the individual peaks with Gaussian functions during recruitment (Figure 1d), the area of each peak versus stimulus voltage is acquired. These data can be plotted as cumulative probabilities, the shapes of which quantify the proportion of the peak recruited at defined stimulus voltages (Figure 1f). The curve fits in Figure 1f provide values of the mean ± SD that were used to express the recruitment data as probability densities (Gaussian distributions) versus stimulus voltage (Figure 1g). The close match of the profiles in Figures 1e with Figure 1g supports the assumption that the axons that produce the 1st peak are the largest, with the lowest recruitment threshold and fastest conduction velocity, whereas the smallest axons with the highest recruitment threshold and slowest conduction velocity produce the 3rd peak.

### 3.2 High‐frequency firing produces a differential decrease in CAP peak amplitudes

To confirm the effects of high‐frequency stimulus on CAP amplitude were not a consequence of Na^+^ channel inactivation we imposed a double pulse protocol in which two sequential supramaximal pulses were applied, with the inter‐pulse interval progressively decreasing (Figure 2a: upper trace). The fidelity of the CAP was maintained until the interval dropped below 5 ms (equivalent to 200 Hz: Figure 2a). These data imply that at stimulus frequencies up to 100 Hz the inter‐pulse interval (10 ms) is sufficient to allow full Na^+^ channel recovery from inactivation. We imposed 4 min periods of stimulus ranging from 5 to 100 Hz. At low frequencies (5–30 Hz), the 3rd peak amplitude decreased at frequencies above 5 Hz, but the 2nd and 1st peaks were more robust, decreasing at higher frequencies (Figure 2b). At higher frequencies (40–100 Hz), the 3rd peak fell whereas the 1st peak was most resilient (Figure 2c,d). On cessation of stimulus, there was a rapid recovery of CAP amplitude, at higher frequencies resulting in overshoot of the CAP, most notable in the 2nd peak (Figure 2c: blue traces). Two main conclusions can be drawn from these results: firstly, as frequency increased the 3rd peak was most susceptible and the 1st peak most resistant to decreased amplitude, and secondly, as the frequency increased the rate of peak decline accelerated.

### 3.3 CAP amplitude varies with aCSF [Na^+^]

We investigated the putative elevations in axonal [Na^+^]_i_ that result from high firing frequency. This process had several steps, the first of which was to quantify the effect of varying aCSF [Na^+^] on CAP amplitude, achieved by decreasing aCSF [Na^+^] sequentially from a baseline of 153 to 30 mM and measuring the amplitude of the CAP peaks in response (Figure 3a). There was no significant difference in amplitude decrease among the 3 peaks, so we used the 2nd peak as representative since it was the largest and most straightforward to measure. We assume the baseline [Na^+^]_i_ was 10 mM (Ransom et al., 2000) and it did not change when aCSF [Na^+^] was reduced. The relationship illustrated in Figure 3b allowed us to predict the CAP amplitude for any aCSF [Na^+^]. As described in the Methods solving this equation in tandem with the Nernst equation allowed us to calculate the *E*_Na_ and [Na^+^]_i_ (Figure 3c), assuming the CAP amplitude is governed exclusively by *E*_Na_. Application of these relationships to each of the three peaks at the range of frequencies imposed generated log linear relationships for [Na^+^]_i_ versus stimulus frequency (Figure 3d).

### 3.4 CAP amplitude varies with aCSF [K^+^]

We determined whether the [K^+^]_o_ levels reached during high‐frequency firing were sufficient to induce Na^+^ channel inactivation, a possible explanation for the reduction in CAP amplitude resulting from high‐frequency firing. This was a two‐step process. Firstly, we used K^+^ sensitive microelectrodes to measure the [K^+^]_o_ increases induced by stimulus. A 2‐min period of 100 Hz led to the characteristic [K^+^]_o_ profile as reported by others in MON (Bay & Butt, 2012). The K^+^ rose rapidly to a peak, followed by a plateau, which was sustained for the duration of the pulse. On cessation of the pulse, the [K^+^]_o_ rapidly fell towards, and then below baseline, before slowly returning towards 3 mM (Figure 4a). The peak [K^+^]_o_ was measured across a wide range of stimulus frequencies (Figure 4b). Secondly, we increased the aCSF [K^+^] in controlled amounts and measured the resulting CAP amplitude. There was no significant difference in the effect among the 3 peaks, so we used the 2nd peak to quantify the effect. The CAP started to fall at 9 mM and was completely lost at 15 mM (Figure 4d).

### 3.5 CAP overshoot and [K^+^]_o_ undershoot

In experiments where MONs were exposed to high stimulus frequency, there was an increase in CAP amplitude, an overshoot, following the stimulus. As this had not been reported previously, we investigated if the phenomenon was related to the [K^+^]_o_ undershoot routinely reported in both white and gray matter following periods of high intensity stimulus (Bay & Butt, 2012; Heinemann & Lux, 1977; Ransom et al., 2000). We simultaneously recorded the [K^+^]_o_ with K^+^ sensitive microelectrodes while recording the CAP. Stimulating the MON for 2 min produced a frequency dependent [K^+^]_o_ undershoot and CAP overshoot. The effect was most prominent in the 2nd peak, which we quantified. The rapid rise in [K^+^]_o_ was not reflected in our measurements of the CAP amplitude profile, which fell comparatively slowly. However on cessation of the stimulus, the decrease in [K^+^]_o_ past baseline, and the overshoot of the CAP mirrored each other closely, suggesting a casual link between low [K^+^]_o_ and the CAP overshoot (Figure 5a). To test this we reduced aCSF [K^+^], which caused an increase in the CAP amplitude. A subsequent 4‐min period of 100 Hz stimulus caused a rapid decrease in the CAP amplitude as previously shown but also a significant overshoot that briefly exceeded the control CAP amplitude before returning to the baseline (Figure 5b). We quantified the [K^+^]_o_ undershoot and CAP overshoot and plotted these on the same graph demonstrating a log linear relationship dependent upon stimulus frequency (Figure 5c), suggesting a causal link between [K^+^]_o_ and CAP amplitude around baseline values of [K^+^]_o_.

## 4 DISCUSSION

The principal result from this study is that large axons in the MON are more resilient to high‐frequency firing than smaller axons, which begin to “run down” during high‐frequency firing due to elevations in axonal [Na^+^]_i_ decreasing *E*_Na_, which attenuates the CAP peak amplitudes, assuming that the action potential peak is governed solely by *E*_Na_. The modest activity‐induced elevations in [K^+^]_o_ are insufficient to impair the CAP (Hille, 2001). The implications of these results relating how energy constraints associated with axon size limit information coding are discussed below.

### 4.1 Energy consumption and information transfer: Size does matter

Encephalization, the process whereby increasing animal size is matched by scaled up brain size (Herculano‐Houzel, 2009), is accompanied by greatly increased energy demands. The human brain contributes only 2% of body weight but consumes 20% of available energy (Hertz & Dienel, 2002). This dramatic disparity between energy consumption and brain (nervous system) size requires compromises in function, in order that space is allocated to most efficiently use the energy resources available (Perge et al., 2009). An obvious example of such a compromise is the relationship between axon size, conduction velocity, and energy consumption (Ju et al., 2016). Axon size in nerve tracts does not scale with animal size, although the number of axons increases accordingly. The human optic nerve tract is 10 times longer than that of the mouse, but the distribution of axons sizes is identical (Perge et al., 2012). Humans forego the increased axon size required for action potential arrival time to match that of the mouse, a trade off between energy saving and decreased responsiveness. The sciatic nerve conducts at 72 ms^−1^ in the elephant and 40 ms^−1^ in the shrew despite the enormous difference in length of the nerves, the loss in agility (compare a lumbering elephant to a scurrying shrew) a favorable energy saving (More et al., 2010). An extreme example of distribution of resources is the decreased visual system in cave dwelling vertebrates, which have traded reduced vision for increased somatosensory processing (Niven & Laughlin, 2008).

Conduction velocity, and thus axon size, can be constrained in all but the most important circuits, for example, proprioception that maintains posture, where the degree of muscle tension is transmitted via primary type 1a sensory fibers, the largest and fastest conducting human axons (Boron & Boulpaep, 2009). However, high‐frequency firing necessitates large axons. The space allocated, energy consumption, and decreased information transfer rate per unit volume are evidence of the importance of this information; increased axon size supports larger terminal arbors where the increased number of active zones facilitates increased synaptic transfer rate of information (Perge et al., 2009). We tested the ability of axons in the MON to conduct at varying frequencies. The MON is an ideal model system as its cylindrical shapes enables CAP recording with suction electrodes (Stys et al., 1991); the presence of a wide range of axon sizes in the same nerve (Allen et al., 2006), which contribute to discrete peaks of the CAP, allows direct inter‐axon comparisons, a considerable advantage over modeling studies.

### 4.2 Stimulus frequency and ion movements

The quadratic relationship of axoplasmic resistance with diameter (Nicholls et al., 2012) dictates that large, myelinated axons exhibit fast conduction and low threshold to stimulus; thus, in our MON model large axons contribute to the 1st peak of the CAP. The close resemblance between the CAP profile and the expression of CAP area versus stimulus voltage as probability densities provides additional supportive evidence. In this present study, changes in the CAP profile reflect changes in individual action potential profile, consistent with the decreases in action potential amplitude and rate of rise when [Na^+^] in seawater bathing squid axons was reduced (Hodgkin & Katz, 1949).

We demonstrated a clear distinction in the response of the CAP peaks to high‐frequency firing. The decline in amplitude denotes the frequency above which the energy demand created by the stimulus exceeds the energy supply. Modeling studies suggest nodal Na^+^ influx as the prime contributing factor in CAP attenuation (Perge et al., 2009; Zang & Marder, 2021). In MON, both experimental and computational studies find small axons fire at 5 Hz whereas large axons fire at 30 Hz, suggesting the principal determinant governing an axon's ability to fire at high frequency is its size (Perge et al., 2009, 2012). If we assume nodal length is fixed at 1 μm and Na^+^ channel density is constant relative to membrane area (Arancibia‐Carcamo et al., 2017), then as axon size increases the number of Na^+^ ions entering the axon for each action potential increases linearly according to πd (*d* = axon diameter), whereas nodal volume increases quadratically as πd^2^, thus the resulting nodal [Na^+^] varies according to 1/*d*. Action potential amplitude is determined by *E*_Na_ (Hodgkin & Katz, 1949), which varies according to the logarithm of the ratio of [Na^+^]_o_/[Na^+^]_i_. It is not technically possible to use imaging techniques to measure [Na^+^]_I_ in neighboring axons of varying diameters in ex vivo MON, so we resorted to indirect methods to deduce reasonable estimates of [Na^+^]_i_ in the three axon populations during high‐frequency firing. The Na^+^ entering the axon during an action potential through TTX sensitive voltage gated channels activates axonal α_3_β_2_ Na^+^K^+^ATPase (NKA), which is insensitive to K^+^ and depolarization, but sensitive to Na^+^ (Larsen et al., 2014; Stanley et al., 2015). It is reasonable to assume that axonal [Na^+^]_i_ increases when available ATP falls below levels required to fuel the NKA, that is, energy supply exceeds demand. However, an alternate explanation is that the K_0.5_ for NKA is at the physiological [Na^+^]_i_, of about 10 mM. Thus, an increase of [Na^+^]_i_ beyond 20 mM, which we predict, would exceed the *V*_max_ of the NKA, limiting the charge translocation capacity of the NKA (Cohen et al., 1984; Holmgren & Rakowski, 2006; Therien & Blostein, 2000). When nodal [Na^+^]_i_ increases, E_Na_ decreases and the CAP peaks decline; thus, attenuation of the peaks signals the frequency above which the axonal NKA is overwhelmed for each axon population. The branch points (see Section 2: Methods) for the 1st and 3rd peaks, 45.5 and 8.9 Hz, respectively, are consistent with the optimal firing frequencies of 30 and 5 Hz reported in large and small axons, respectively (Perge et al., 2009). These estimates also agree with the frequencies at which [Na^+^]_i_ exceeds 15 mM in the 1st and 3rd peak axons, 32.3 and 7.2 Hz, respectively. Supporting evidence comes from sciatic nerve studies where the A peak, produced by large myelinated axons up to 15 μm in diameter (Rich & Brown, 2018), can conduct at stimulus frequencies of 100 Hz (Rich et al., 2022). The proposed decline in the small axon conduction during high‐frequency firing predicted from modeling studies (Zang & Marder, 2021) has recently been challenged. The model used Ohm's law to describe *I*_Na_, which led to loss of small axon conduction. However using the Goldman–Hodgkin–Katz current equation (Hille, 2001) led to smaller *I*_Na_ at depolarized membrane potentials, which supports small axon action potential conduction despite increases in [Na^+^]_i_ (Kotler et al., 2022). Our results do not support this conclusion.

The dogmatic view of K^+^ efflux via voltage gated delayed rectifier (K_V_) K^+^ channels is unlikely to occur in central myelinated axons. Immunohistochemical studies have localized K_V_ channels to the juxtaparanodal regions (Rasband & Shrager, 2000); thus, any K^+^ efflux would accumulate in the submyelin space, isolated from the interstitial fluid by tight junctions (Devaux & Gow, 2008; Gow & Devaux, 2008). A more likely route of K^+^ efflux is via large conductance two‐pore domain (K_2P_) K^+^ leak channels (Schwarz, 2021) located at the nodes, where K^+^ fluxes respond rapidly to membrane potential changes as dictated by the Goldman–Hodgkin–Katz equation (Brohawn et al., 2019; Schwarz, 2021). The membrane depolarization caused by Na^+^ influx during an action potential would be rapidly countered by K^+^ efflux into the nodal region, which is supported by recordings of intracellular action potentials from rat optic nerve axons, which lack an after‐hyperpolarization (Gordon et al., 1988). Activity dependent K^+^ efflux accumulates in the restricted interstitial space, but we only recorded an increase of about 1.5 mM, in agreement with previous MON studies (Bay & Butt, 2012), although studies in rat optic nerve show ceiling levels of 8 mM (Ransom et al., 2000), still insufficient to significantly inactivate Na^+^ channels. The disparity in the magnitude of stimulus evoked [K^+^]_o_ elevations between mouse and rat may be explained by the small size of the MON limiting the separation of the interstitial fluid pool at the tip of the K^+^‐sensitive electrode and the bath aCSF. Any changes in interstitial [K^+^] would be attenuated by the rapid exchange between the two compartments, a situation that would not arise in the larger RON. Astrocytes express α_2_β_2_ NKA, which is stimulated by elevations in interstitial [K^+^] and the associated membrane depolarization, but is insensitive to Na^+^ (Larsen et al., 2014; Stanley et al., 2015). In addition, astrocytes express the constitutively open Kir4.1 channels, which act as routes of K^+^ entry and efflux (MacAulay, 2020), and maintain a hyperpolarized membrane potential. Spatial buffering of astrocytic K^+^ is achieved where K^+^ influx via Kir4.1 channels is counteracted by electrotonic repulsion of K^+^ from distant astrocytes in the interconnected syncytium (Barros, 2022; Orkand et al., 1966). The combination of these transport mechanisms is computed as the most energy efficient means of buffering interstitial K^+^ (Barros, 2022). Astrocytes occupy 30% of the volume of the MON and contain 70% of the mitochondria (Perge et al., 2009). Astrocytes are extremely efficient at buffering interstitial K^+^, such that during physiological activity [K^+^]_o_ never exceeds a celling level at which action potentials can propagate without loss of fidelity. Thus, we are confident that excessive K^+^ accumulation does not contribute to CAP attenuation during high‐frequency firing.

**FIGURE 6:**
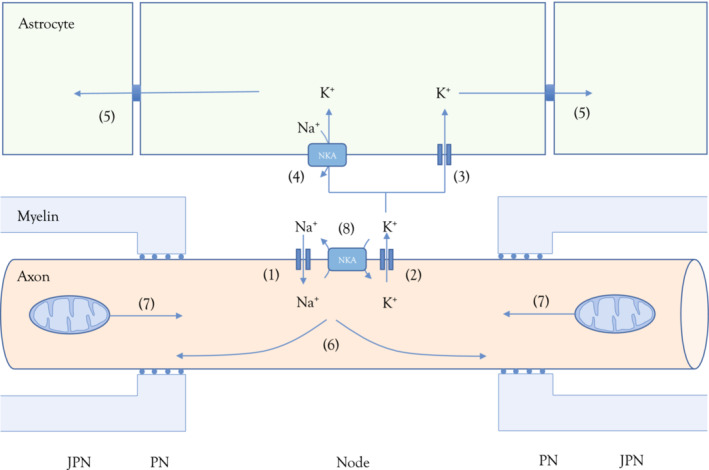
Stimulus induced trans‐membrane ion movements. Axonal action potentials are produced by Na^+^ influx via TTX sensitive voltage gated Na^+^ channels (1) with K^+^ leaving via nodal K_2P_ channels (2). Constitutively open astrocytic Kir4.1 channels (3) and the NKA (4) buffer the elevated interstitial K^+^, which causes electrotonic repulsion of K^+^ from distant astrocytes in the syncytium (5), an example of spatial buffering (MacAulay, 2020). The stimulus induced elevation in nodal Na^+^ is predicted to spread laterally (6) to (juxta)paranodal regions (Zang & Marder, 2021). Mitochondria in these regions are sensitive to elevated Na^+^ and can move towards the nodes (7) to facilitate the axonal NKA in restoring baseline [Na^+^] (Ohno et al., 2011).

However, we report a post‐stimulus decrease in [K^+^]_o_ below baseline, the undershoot, a feature of both gray and white matter (Bay & Butt, 2012; Heinemann & Lux, 1977; Ransom et al., 2000). A likely mechanism is that effective K^+^ buffering maintains [K^+^]_o_ at a low level during sustained firing, while axonal [Na^+^]_i_ steadily increases (Figure 6). On cessation of stimulus the imbalance between axonal Na^+^ accumulation and stable [K^+^]_o_, where electro‐neutrality dictates that K^+^ entry matches Na^+^ efflux, leads to axonal K^+^ uptake via activation of the axonal NKA by increased [Na^+^]_i_ that drives [K^+^]_o_ below baseline levels. The low [K^+^]_o_ leads to axonal hyperpolarization, causing priming of inactivated Na^+^ channels, which contribute to subsequent action potentials thereby augmenting the CAP amplitude.

## 5 CONCLUSION

Our data provide evidence of discrete effects of the trans‐membrane ion movements that occur during high‐frequency firing. Whereas small axon conduction fails rapidly when faced with high‐frequency firing, large axons are more resilient and are thus the ideal conduit for the transmission of high‐frequency information. In small axons, rapid elevations in nodal [Na^+^] result from Na^+^ entry, reducing E_Na_ thereby decreasing the CAP. The resilience of large axons to the metabolic effects of high‐frequency firing emphasizes the consequences of volume specific scaling of energy expenditure (Karbowski, 2007). However, efficient astrocytic K^+^ buffering imposes a ceiling level on [K^+^]_o_, where K^+^ acts as a signaling mechanism (Bittner et al., 2011; Sotelo‐Hitschfeld et al., 2015) informing astrocytes via activation of the NBC co‐transporter (Ruminot et al., 2011, 2019) of on‐going neuronal activity and an immediate energy requirement.

## AUTHOR CONTRIBUTIONS

AMB was responsible for the conception and design of the study. AJH, HBJ, and AMB carried out all the electrophysiology studies. AJH and HBJ carried out the K^+^‐sensitive microelectrode studies. AJH and HBJ analyzed the data. AMB wrote the paper with input from AJH and HBJ. All authors approved the final version of the work submitted for publication and agree to be accountable for all aspects of this work.

## FUNDING INFORMATION

This research did not receive any specific grant from funding agencies in the public, commercial, or not‐for‐profit sectors. All costs were covered by institutional funding from the University of Nottingham.

## CONFLICT OF INTEREST STATEMENT

None declared.
